# Clusters of social and substance use-related risks are associated with the duration of untreated psychosis

**DOI:** 10.1017/S0033291726103791

**Published:** 2026-05-05

**Authors:** Hannah Edelhoff, Jim van Os, Therese van Amelsvoort, Claudia J. P. Simons, Lieuwe de Haan, Marieke van der Pluijm, GROUP Investigators, Lucia Sideli, Ilaria Tarricone, Laura Ferraro, Sarah Tosato, Domenico Berardi, Celso Arango, Miguel Bernardo, Paulo Rossi Menezes, Cristina Marta Del-Ben, Franck Schürhoff, Jean-Paul Selten, Bart P. F. Rutten, Robin M. Murray, Craig Morgan, Frederike Schirmbeck, Ulrich Reininghaus

**Affiliations:** 1 Department of Public Mental Health, Central Institute of Mental Health, Medical Faculty Mannheim, University of Heidelberg, Mannheim, Germany; 2 Department of Psychiatry, Utrecht University Medical Center, Utrecht, The Netherlands; 3 Department of Psychiatry and Neuropsychology, School for Mental Health and Neuroscience, Maastricht University Medical Centre, Maastricht, The Netherlands; 4 Department of Psychosis Studies, Institute of Psychiatry, Psychology & Neuroscience, King’s College London, London, UK; 5 Mental Health and Neuroscience Institute, Maastricht University, Maastricht, The Netherlands; 6 Department of Psychiatry and Neuropsychology, Maastricht University Medical Center, Maastricht, The Netherlands; 7 GGzE Institute for Mental Health Care, Eindhoven, The Netherlands; 8 Department of Psychiatry, Early Psychosis Section, Amsterdam Medical Center, University of Amsterdam, Amsterdam, The Netherlands; 9 Department of Research, Arkin Mental Health Care, Amsterdam, The Netherlands; 10 Department of Child and Adolescent Psychiatry, Institute of Psychiatry and Mental Health, IiSGM, CIBERSAM, Hospital General Universitario Gregorio Marañón, Madrid, Spain; 11 Department of Medical and Surgical Science, Psychiatry Unit, Alma Mater Studiorum, University of Bologna, Bologna, Italy; 12 Department of Biomedicine, Neuroscience and Advanced Diagnostics, Section of Psychiatry, University of Palermo, Palermo, Italy; 13 Department of Neuroscience, Biomedicine and Movement Sciences, Section of Psychiatry, University of Verona, Verona, Italy; 14 Department of Medical and Surgical Science, Psychiatry Unit, University of Bologna, Bologna, Italy; 15 Barcelona Clínic Schizophrenia Unit, Hospital Clínic, Institut d’Investigacions Biomèdiques August Pi I Sunyer (IDIBAPS), University of Barcelona, Barcelona, Spain; 16 Department of Preventive Medicine, Faculty of Medicine, University of São Paulo, São Paulo, Brazil; 17 Department of Neuroscience and Behavior, Ribeirão Preto Medical School, University of São Paulo, São Paulo, Brazil; 18 INSERM, IMRB, AP-HP, Hôpitaux Universitaires “H. Mondor”, DMU IMPACT, Fondation Fondamental, 94010, University Paris-Est Créteil Val de Marne, Créteil, France; 19 Department of Psychiatry and Neuropsychology, School for Mental Health and Neuroscience, South Limburg Mental Health Research and Teaching Network, Maastricht University Medical Centre, MD Maastricht, Maastricht, The Netherlands; 20 Institute of Psychiatry, Psychology and Neuroscience, King’s College London, London, UK; 21 Centre for Epidemiology and Public Health, Health Service and Population Research Department, Institute of Psychiatry, Psychology & Neuroscience, King’s College London, London, UK; 22 ESRC Centre for Society and Mental Health, King’s College London, London, UK; 23 Health Service and Population Research Department, Institute of Psychiatry, Psychology & Neuroscience, King’s College London, London, UK; 24 German Center for Mental Health (DZPG), partner site Mannheim-Heidelberg-Ulm, Germany

**Keywords:** health inequalities, latent class analysis, schizophrenia

## Abstract

**Background:**

The duration of untreated psychosis (DUP) is still considerably long in patients with psychotic disorders worldwide. Social determinants, such as the socioeconomic status, can influence DUP, exacerbating health inequalities in access to timely care. We investigated whether subpopulations with shared characteristics are associated with longer DUP.

**Methods:**

We performed latent class analyses to investigate whether classes with shared configurations of social and substance use-related risks can be identified in two large cohorts with psychotic disorders: *N* = 780 patients from the GROUP project and *N* = 847 patients from the EU-GEI project. Subsequently, we conducted survival analyses to analyze whether identified classes are associated with DUP.

**Results:**

We identified three classes in both samples. Membership of the class with predominantly younger men, higher proportion of cannabis use, and supported living was associated with longer DUP compared with a class with predominantly White ethnicity, higher education, and current employment in GROUP (HR = 1.28, 95% CI: 1.06–1.56, *p* = .011) and in EU-GEI (HR = 1.27, 95% CI: 1.07–1.51, *p* = .007). In GROUP, membership of a third class with predominantly White women, without cannabis use, was associated with the shortest DUP (HR = 0.78, 95% CI: 0.63–0.95, *p* = .016).

**Conclusions:**

Results suggest that specific populations differ in their risk distributions for prolonged DUP and highlight the importance of considering configurations of social determinants in context. Public mental health programs need to establish their differential impact for diverse populations and facilitate more targeted pathways to care.

## Introduction

Social determinants of health and health inequalities in patients with psychotic disorders have gained increasing attention over the past decades (Jester et al., 2023; Kirkbride et al., 2024). Lower socioeconomic status (SES) and migration status are thought to be associated with higher rates of psychotic disorders (Jester et al., 2023). Simultaneously, they impact the duration of untreated psychosis (DUP), the time between disorder onset and initiation of treatment (Singh et al., 2005), a key predictor for symptom severity, remission, and overall functioning (Howes et al., 2021). Despite efforts to implement early detection and intervention, DUP is still considerably long (*M* = 42.6 weeks, 95% CI = 40.6–44.6) worldwide (Salazar de Pablo et al., 2024). Hence, we still need to understand more fully how social determinants delay treatment initiation in potentially marginalized and underserved groups.

SES, as one of the most widely studied determinants, has consistently been found to be associated with DUP (Fordham et al., 2023; Peralta et al., 2005). More specifically, lower levels of educational attainment (Skrobinska, Newman-Taylor, & Carnelley, 2024; Souaiby et al., 2019; Takizawa et al., 2021) and unemployment (Limbu, Nepal, & Mishra, 2025; Morgan et al., 2006; Qiu et al., 2019; Reichert & Jacobs, 2018), two key indicators of lower SES, were associated with longer DUP. However, in a study from the United Kingdom, the association with unemployment was only found when patients also reported lower levels of social contacts, which might point to a protective effect of social contacts (Reininghaus et al., 2008). In this line, patients living with family members (Compton et al., 2011) or those reporting family involvement in help-seeking (Morgan et al., 2006) had, on average, shorter DUP. Further, ethnic and migrant minority group status was linked to longer DUP, though it is critical to consider the local context (Boonstra et al., 2012; Schoer, Huang, & Anderson, 2019). In addition, female gender (Skrobinska et al., 2024) has been linked with earlier help-seeking in psychosis.

In sum, findings suggest that social factors seem to be associated with a longer DUP. So far, most studies have investigated the role of one or two factors independently (Jester et al., 2023), without taking into account that effects might unfold differently depending on the broader (social) context. This might, for example, explain the conflicting results for cannabis use, which was found to be associated with shorter DUP (Burns, 2012) and also longer DUP in patients with an early disorder onset, whereas no association was observed for alcohol and tobacco use (Fond et al., 2018). The impact of certain variables might differ between subpopulations, so that the same factor (e.g., cannabis use) can delay and accelerate treatment initiation depending on context and population. Moreover, shared configurations of certain factors may contribute to uneven risk distributions that put marginalized populations at higher risk of exposure to further risks and negative outcomes (Reininghaus et al., 2024). To our knowledge, only one cohort study in the United States investigated clusters of several determinants together: the most disadvantaged groups in terms of social position and first-contact (e.g. homelessness, language fluency, and emergency contacts) had the longest time to first contact, whereas the more advantaged groups (e.g. private insurance, predominantly White, and outpatient service use) had the shortest (van der Ven et al., 2022). These results imply that risk may cluster within specific subgroups sharing similar configurations of determinants, which, in turn, may increase or decrease risk for prolonged DUP, respectively. Improving our understanding of the effects of social and substance use-related risks in accessing adequate care may help inform early intervention to reduce, rather than augment, socioeconomic inequities in (mental) health. Therefore, the current study aimed to: (a) investigate whether different classes of patients with psychosis can be identified that share similar configurations of social and substance use-related risks (i.e. sex, ethnicity, cannabis use, employment, education, and living situation) and (b) examine whether identified classes are associated with DUP. Individuals with several potential risk factors (e.g. ethnic minority group and lower educational attainment) are posited to reflect a particularly vulnerable subpopulation for prolonged DUP.

## Material and methods

### Study design and participants

The current study conducted parallel analyses in two large samples to descriptively compare the identified clusters: first, in patients with psychotic disorders recruited within the Genetic Risk and Outcome of Psychosis (GROUP) Study, and second, in patients with first-episode psychosis (FEP) from the European Network of National Schizophrenia Networks Studying Gene–Environment Interactions (EU-GEI). GROUP was a naturalistic longitudinal cohort study that recruited patients with psychotic disorders, relatives, and controls from four sites in the Netherlands and the Dutch-speaking part of Belgium in 2004–2008 (Korver et al., 2012). Inclusion criteria for patients comprised aged 16–50 years, lifetime nonaffective psychotic disorder, Dutch language proficiency, and the ability to give informed consent. Baseline data from release 8.0 of the GROUP database were used for the current analyses. EU-GEI was a population-based incidence and case–control study that recruited patients with FEP and controls across 17 sites in 6 countries (United Kingdom, the Netherlands, France, Spain, Italy, and Brazil) in 2010–2015 (Gayer-Anderson et al., 2020). The inclusion criteria for patients comprised age between 18 and 64 years, residency in a catchment area at first presentation, and a first episode of an affective or nonaffective psychotic disorder. Exclusion criteria for both studies were organic causes of psychotic symptoms, symptoms due to acute intoxication, and intellectual disabilities. Additionally, EU-GEI excluded participants with prior contact with specialist mental health services for psychotic symptoms. GROUP was approved by the Medical Ethics Committee of the Academic Medical Center of Utrecht, and EU-GEI was approved by the respective ethics committees from each site (Jongsma et al., 2018). For a detailed description of the study design and population, please refer to the study protocols (Gayer-Anderson et al., 2020; Korver et al., 2012).

### Measures

Sociodemographic characteristics were assessed with the modified Medical Research Council sociodemographic schedule (Mallett, 1997) in both studies. Sex was dichotomized into men and women. Ethnicity was dichotomized into White and ethnic minority (GROUP: Moroccan, Surinamese, Turkish, Antillean, Asian, Mixed, and Other; EU-GEI: Black, Mixed, Asian, North African, and Other). Disaggregation by ethnicity was not possible due to the small sample sizes of each ethnic group. Employment was categorized into unemployed, part-time (including students), and full-time (including self-employed). Education was harmonized between countries into no school-leaving education/elementary, secondary, and university level. Living situation was defined based on prior work (Poppe et al., 2024) and categorized into supported living (sheltered living/with parent(s)), individual living (with partner/family), and living alone.

Cannabis use in the past 12 months was assessed using the Composite International Diagnostic Interview (World Health Organization, 1994) in GROUP and the modified Cannabis Experience Questionnaire (Di Forti et al., 2009) in EU-GEI. Both instruments use a dichotomous coding scheme of 1 = yes and 0 = no for cannabis use.

The DUP was assessed using the Life Chart Schedule (Sartorius et al., 1996) in GROUP and the Nottingham Onset Schedule (Singh et al., 2005) in EU-GEI (see Supplementary Material for details on interview training and assessment). In GROUP, DUP was defined as the time between the first psychotic episode, in which hallucinations, delusions, or disorganized speech or thinking were present for at least 1 week, and the start of antipsychotic medication and/or first contact with mental health professionals for psychosis. It was originally assessed in months and transformed to weeks to improve interpretability and comparability between the samples. In EU-GEI, the DUP was defined as the number of weeks from the first positive psychotic symptom to the initiation of antipsychotic treatment.

### Missing data and outlier analysis

We analyzed the distribution of missing data (Harrison & Riinu, 2020) and visualized the patterns of missingness with the packages ‘naniar’ (Tierney & Cook, 2023) and ‘finalfit’ (Harrison, Drake, & Pius, 2024). To improve the interpretability of identified classes, we choose to only include participants with complete data on all indicator variables in both samples. Moreover, outliers on the duration of illness (DUI) in GROUP were investigated and excluded (see Table 3). Regarding between-class comparisons on the time to treatment, individuals with missing values were excluded, and outliers were investigated (see Tables 3 and 5).

### Statistical analysis

The overall analytic plan was preregistered, with subsequent updates made, including the addition of the EU-GEI sample and alterations to the models based on observed patterns of missing data (Edelhoff, GROUP Contributors, EU-GEI Contributors, & Schirmbeck, 2024). Latent class analysis (LCA) was used to identify subgroups of patients with psychosis endorsing a similar set of social and substance use-related risks in GROUP and EU-GEI patients separately. We included sex, ethnicity, cannabis use, employment, education, and living situation as categorical indicators in the LCA. All analyses were conducted using RStudio version 4.2.2 (R Core Team, 2022). LCA models were fit with the ‘poLCA’ package (Linzer & Lewis, 2011). Following statistical and methodological guidelines (Geiser, 2011; Nylund, Asparouhov, & Muthén, 2007; Sinha, Calfee, & Delucchi, 2021), the number of classes was determined based on Bayesian Information Criterion (BIC), sample-size adjusted BIC (aBIC), and consistent Akaike Information Criterion (cAIC), with lower values indicating better model fit. For statistical model comparison, we focused on BIC and aBIC indices, as they are considered the best indices, especially in large samples (Nylund et al., 2007). In addition, we investigated relative entropy, the conditional item response probabilities, and took the relative number of cases per class into account (Sinha et al., 2021). For final decisions on class solution, we strongly considered which solution best described the variability in the datasets in terms of distinctiveness, so either high or low levels in the indicator variables, but not medium (Geiser, 2011), which is key for interpretability and usefulness of the LCA. For subsequent analysis, individuals were classified according to their most likely class membership using posterior probabilities. Associations between class membership and time to treatment initiation were investigated using Cox proportional hazard regression analyses using the ‘survival’ package (Therneau, 2024). Kaplan–Meier plots were created with ‘ggsurvfit’ (Sjoberg et al., 2024) to visualize the time to treatment.

## Results

### Sample characteristics

Initially, GROUP recruited 1,119 patients with psychotic disorders and EU-GEI recruited 1,130 patients with FEP. After visual inspection, there were no indicators for values not missing at random (see Figures S1–S4). As can be seen in Table 1, the participants in GROUP were on average 27 years old (SD = 7.18), with the majority being men and identifying as White ethnicity. The participants in EU-GEI were on average 31 years old (SD = 10.74), the sex distribution was more balanced with 61% men, and the majority identified as White ethnicity. In both samples, schizophrenia was the most frequent diagnosis.

**Table 1.** Basic sample characteristics of GROUP and EU-GEI

Variable	GROUP sample (*n* = 780)^a^	EU-GEI sample (*n* = 847)^b^
Age (in years), *M* (SD)	26.84 (7.18)	31.21 (10.74)
Sex, male, *n* (%)	606 (77.7)	512 (60.5)
Ethnicity, *n* (%)		
*White*	623 (79.9)	550 (64.9)
*Black*	–	127 (15.0)
*North African*	–	30 (3.5)
*Moroccan*	18 (2.31)	–
*Surinamese*	16 (2.05)	–
*Turkish*	13 (1.67)	–
*Antillean*	4 (0.51)	–
*Asian*	2 (0.26)	27 (3.2)
*Mixed*	83 (10.64)	91 (10.7)
*Other*	21 (2.69)	22 (2.6)
Cannabis use, yes, *n* (%)	291 (37.3)	176 (20.8)
Highest education, *n* (%)		
*No education/elementary*	104 (13.3)	139 (16.4)
*Secondary*	577 (74.0)	578 (68.2)
*University*	99 (12.7)	130 (15.4)
Employment status, *n* (%)		
*Unemployed*	338 (43.3)	344 (40.6)
*Part-time*	230 (29.5)	186 (22.0)
*Full-time*	212 (27.2)	317 (37.4)
Living situation, *n* (%)		
*Alone*	265 (34.0)	144 (17.0)
*Supported (parent(s), sheltered)*	433 (55.5)	364 (43.0)
*Individual (partner, family)*	82 (10.5)	339 (40.0)
Diagnosis, *n* (%)^c^		
*Schizophrenia*	516 (66.2)	408 (48.3)
*Schizoaffective*	97 (12.4)	46 (5.4)
*Depressive*	–	129 (15.3)
*Manic, bipolar*	–	132 (15.6)
*Delusional disorder*	18 (2.3)	43 (5.1)
*NOS, other, and uncertain* ^d^	148 (19.0)	87 (10.3)
DUP antipsychotics (in weeks)^e^		
*M (SD)*	33.98 (19.38)	29.66 (60.58)
*Median (IQR)*	4.33 (4.33–39)	6 (6–20)
DUP mental health contact (in weeks)^f^		
*M (SD)*	25.92 (52.27)	
*Median (IQR)*	0 (0–21.67)	–

a *n* = 311 participants were excluded due to missing values and another *n* = 28 outliers with duration of illness values exceeding 2 SDs above the mean were excluded;

b *n* = 283 participants were excluded due to missing values;

c missing diagnosis: *n* = 1 (0.1%) in GROUP and *n* = 2 (0.2%) in EU-GEI;

d NOS = not otherwise specified psychosis; this category includes uncertain diagnosis and cases where the algorithm could not derive diagnosis;

e *n* = 104 (13.3%) participants (in GROUP) and *n* = 82 (9.7%) participants (in EU-GEI) were excluded from the between-class comparisons, see Tables 3 and 5, as well as methods section for more details;

f *n* = 109 (14.0%) participants were excluded from the between-class comparisons in GROUP.

### Identified classes of social determinants and their association with DUP in GROUP

The goodness-of-fit indices in GROUP are presented in Table 2. The BIC and aBIC were lowest for the two-, three-, and four-class solution. The four-class solution was discarded, as the smallest class was only 2.1% of the sample, which is lower than the recommended 5–8% (Nylund-Gibson & Choi, 2018) and indicates overextraction (Sinha et al., 2021). Moreover, the relative entropy increased in the four-class solution, indicating more uncertainty in the classification. Overall, the goodness-of-fit indices mostly favored the two-class solution, though the aBIC was the lowest in the three-class solution. We then compared interpretability and distinctiveness. The two-class solution was more difficult to interpret (see Supplementary Table S1), reflecting in no clear predominance for either of the categories (e.g. employment and living situation). The three-class solution had clearer patterns, especially for sex and cannabis use (most prominent in class 1), and for education and living situation (most visible in classes 2 and 3). Overall, the three-class solution showed lightly lower aBIC, comparable entropy, sufficient size of all extracted classes, and superior interpretability, and, hence, was selected as the best-fitting solution.

**Table 2.** Indices of the latent class analysis in GROUP for 1–6 classes

No. class	Log-likelihood	Resid. df	BIC^a^	aBIC^b^	cAIC^c^	Likelihood-ratio	Relative entropy^d^	Smallest class, *n* (%)
1	−3474.13	206	7008.20	6979.62	7017.20	405.41	–	–
2	−3402.74	196	6932.01	6871.68	6951.01	262.63	0.53	196 (25.1)
3	−3374.44	186	6942.00	6849.91	6971.00	206.03	0.59	133 (17.1)
4	−3361.98	176	6983.67	6859.83	7022.67	181.10	0.71	16 (2.1)
5	−3348.84	166	7023.98	6868.38	7072.98	154.82	0.69	70 (9.0)
6	−3338.25	156	7069.39	6882.04	7128.39	133.63	0.80	18 (2.3)

a Bayesian information criterion;

b sample size-adjusted Bayesian information criterion;

c consistent Akaike information criterion;

d higher values equal higher uncertainty in classification.

Descriptive statistics for social and substance use-related variables for the total GROUP sample and broken down by the three classes are shown in Table 3. The following is a description of the most prominent indicator variables per class.

**Table 3.** Descriptive statistics of social and substance use-related risks and DUP for the total sample and three classes in GROUP

Variable	Total sample (*n* = 780)	Class 1 (*n* = 133, 17.0%)	Class 2 (*n* = 474, 60.7%)	Class 3 (*n* = 173, 22.2%)
Age (in years), *M* (SD)	26.84 (7.18)	29.37 (8.79)	24.67 (5.96)	30.84 (6.52)
Sex, male, *n* (%)	606 (77.7)	0 (0)	438 (92.4)	168 (97.1)
Ethnicity, *n* (%)				
*White*	623 (79.9)	123 (92.5)	331 (69.8)	169 (97.7)
*Moroccan*	18 (2.31)	0 (0)	18 (3.8)	0 (0)
*Surinamese*	16 (2.05)	1 (0.8)	15 (3.2)	0 (0)
*Turkish*	13 (1.67)	1 (0.8)	12 (2.5)	0 (0)
*Antillean*	4 (0.51)	1 (0.8)	3 (0.6)	0 (0)
*Asian*	2 (0.26)	1 (0.8)	1 (0.2)	0 (0)
*Mixed*	83 (10.64)	4 (3.0)	75 (15.8)	4 (2.3)
*Other*	21 (2.69)	2 (1.5)	19 (2.7)	0 (0)
Cannabis use, yes, *n* (%)	291 (37.3)	0 (0)	226 (47.7)	65 (37.7)
Highest education, *n* (%)				
*No education/elementary*	104 (13.3)	3 (2.3)	101 (21.3)	0 (0)
*Secondary*	577 (74.0)	95 (71.4)	376 (77.4)	115 (66.5)
*University*	99 (12.7)	35 (26.3)	6 (1.3)	58 (33.5)
Employment status, *n* (%)				
*Unemployed*	338 (43.3)	65 (48.9)	257 (54.2)	16 (9.2)
*Part-time*	230 (29.5)	29 (21.8)	111 (23.4)	90 (52.0)
*Full-time*	212 (27.2)	39 (29.3)	106 (22.4)	67 (38.7)
Living situation, *n* (%)				
*Alone*	265 (34.0)	35 (26.3)	103 (21.7)	127 (73.4)
*Supported (parent(s), sheltered)*	433 (55.5)	61 (45.9)	359 (75.7)	13 (7.5)
*Individual (partner, family)*	82 (10.5)	37 (27.8)	12 (2.5)	33 (19.1)
DUP antipsychotics (in weeks)^a^				
*M (SD)*	33.98 (19.38)	26.37 (48.80)	39.23 (65.80)	24.94 (24.94)
*Median (IQR)*	4.33 (4.33–39)	4.33 (4.33–26.00)	8.67 (8.67–43.30)	4.33 (4.33–32.50)
DUP mental health contact (in weeks)^b^				
*M (SD)*	25.92 (52.27)	15.04 (35.47)	30.12 (57.72)	22.88 (45.92)
*Median (IQR)*	0 (0–21.67)	0 (0–13.00)	4.33 (4.33–27.10)	0 (0–20.60)

a We excluded *n* = 104 (13.3%) participants from the between-class comparisons on time to treatment, thereof *n* = 84 due to missing values and *n* = 20 for outlier status (values above 3 SDs for DUP); *n* = 16 (12.0%) in class 1, *n* = 58 (12.2%) in class 2, and *n* = 30 (17.3%) in class 3.

b we excluded *n* = 109 (14.0%) participants from the between-class comparisons on time to treatment, thereof *n* = 86 due to missing values and *n* = 23 for outlier status (values above 3 SDs for DUP); *n* = 16 (12.0%) in class 1, *n* = 66 (13.9%) in class 2, and *n* = 27 (15.6%) in class 3.

#### Class 1: White women without cannabis use and higher unemployment

Class 1 was the smallest class, comprising female (100%), predominantly White (92.5%) patients with no cannabis use (0%), and a higher level of educational attainment (71.4% completed secondary education, 26.3% with university degree). Almost half of the class (48.9%) was unemployed at time of assessment.

#### Class 2: Younger men, with lower educational attainment, higher unemployment, cannabis use, and supported living situation

Class 2 was the largest class with predominantly men (92.4%) and comparably younger age (*M* = 24.67 years, SD = 5.96). This class had the highest proportions of patients from ethnic minority groups (30.2%). Almost half of the patients reported cannabis use (47.7%). Patients had comparably lower levels of educational attainment (21.3% without a school-leaving qualification/elementary). Half of the patients were unemployed at the time of assessment (54.2%), and the majority lived in supported housing, that is, with parent(s) or in sheltered accommodations (75.7%).

#### Class 3: White men, employed, with higher educational attainment, often living alone

Patients in class 3 were on average 30.84 years old (SD = 6.53), predominantly male (97.1%), and White (97.7%), and most of them were living alone (73.4%) at the time of assessment. The patients completed secondary education (66.5%) or a higher educational level (33.5%), and most of them were working part-time/were students (52.0%) or full-time (38.7%) at the time of assessment.

Findings from Cox proportional hazard regression analysis for DUP before antipsychotic treatment indicated a shorter time to treatment in class 1 compared with class 2 (HR = 0.78; 95% CI: 0.63–0.95, *p* = .016). Class 2 had a longer time to antipsychotic treatment compared with class 3 (HR = 1.28; 95% CI: 1.06–1.56, *p* = .011). No differences were found between class 1 and class 3 (HR = 0.99, 95% CI 0.78–1.27, *p* = .978). For DUP mental health contact, class 1 showed a shorter time to treatment compared with class 2 (HR = 0.71; 95% CI: 0.58–0.88, *p* = .001). No differences in DUP were found for comparisons between class 1 and 3 (HR = 0.82, 95% CI 0.64–1.05, *p* = .110), or class 2 and 3 (HR = 1.15, 95% CI 0.95–1.39, *p* = .150). The respective Kaplan–Meier plots are shown in Figures S5 and S6 in the Supplementary Material. Proportional hazard assumption was inspected via Schoenfeld residuals and given for both DUP antipsychotics *χ*^2^(2, *N* = 676) = 2.06, *p* = .36 and DUP mental health contact *χ*^2^(2, *N* = 671) = 0.08, *p* = .96, indicating that the hazard ratios are constant over time.

### Identified classes of social determinants and their association with DUP in EU-GEI

The goodness-of-fit indices in EU-GEI are presented in Table 4. BIC was lowest for the two- and three-class solution, and aBIC was lowest for the three- and four-class solution. Although the aBIC remained the same, the entropy increased considerably for the four-class solution, indicating more uncertainty in classification, and this solution was therefore discarded. Overall, the statistical goodness-of-fit indices favored the two-class solution. We compared the interpretability and distinctiveness of the solutions. The two-class solution (see Supplementary Table S2) had a less clearly interpretable pattern, reflected in predominance for some of the characteristics, whereas in the three-class solution, sex, ethnicity, and employment (class 3 vs. class 1/2) were more clearly distributed across the classes. Overall, the three-class solution showed low BIC and aBIC, good entropy, sufficient class sizes, and superior interpretability, and hence was selected as the best-fitting solution.

**Table 4.** Indices of the latent class analysis in EU-GEI for 1–6 classes

No. class	Log-likelihood	Resid. df	BIC^a^	aBIC^b^	cAIC^c^	Likelihood-ratio	Relative entropy^d^	Smallest class, *n* (%)
1	−4042.21	206	8145.09	8116.51	8145.09	418.06	–	–
2	−3992.47	196	8112.03	8052.69	8132.03	318.59	0.42	345 (40.73)
3	−3959.13	186	8113.77	8021.67	8142.77	251.90	0.43	255 (30.11)
4	−3941.25	176	8145.43	8021.58	8184.43	216.16	0.60	142 (16.76)
5	−3929.19	166	8188.72	8033.11	8237.72	192.02	0.59	72 (8.50)
6	−3917.84	156	8233.44	8046.08	8292.44	169.33	0.63	70 (8.26)

a Bayesian information criterion;

b sample size-adjusted Bayesian information criterion;

c consistent Akaike information criterion;

d higher values equal higher uncertainty in classification.

Descriptive statistics of indicator variables for the social and substance use-related risks by the total sample and broken down by the three classes are shown in Table 5.

**Table 5.** Descriptive statistics of social and substance use-related risks, DUP, and clinical characteristics for the total sample and classes in the three-class solution in EU-GEI

Variable	Total sample (*n* = 847)	Class 1 (*n* = 255, 30.1%)	Class 2 (*n* = 320, 37.8%)	Class 3 (*n* = 272, 32.1%)
Age (in years), *M* (SD)	31.21 (10.74)	34.73 (11.17)	26.36 (8.32)	33.63 (10.80)
Sex, male, *n* (%)	512 (60.5)	101 (39.6)	313 (97.8)	98 (36.0)
Ethnicity, *n* (%)				
*White*	550 (64.9)	92 (36.1)	204 (63.8)	254 (93.4)
*Black*	127 (15.0)	66 (25.9)	50 (15.6)	11 (4.0)
*North African*	30 (3.5)	16 (6.3)	13 (4.1)	1 (0.4)
*Asian*	27 (3.2)	14 (5.5)	12 (3.8)	1 (0.4)
*Mixed*	91 (10.7)	53 (20.8)	34 (10.6)	4 (1.5)
*Other*	22 (2.6)	14 (5.5)	7 (2.2)	1 (0.4)
Cannabis use, yes, *n* (%)	176 (20.8)	33 (12.9)	121 (37.8)	22 (8.1)
Highest education, *n* (%)				
*No education/elementary*	139 (16.4)	93 (36.5)	46 (14.4)	0 (0)
*Secondary*	578 (68.2)	129 (50.6)	273 (85.3)	176 (64.7)
*University*	130 (15.4)	33 (12.9)	1 (0.3)	96 (35.3)
Employment status, *n* (%)				
*Unemployed*	344 (40.6)	174 (68.2)	132 (41.2)	38 (14.0)
*Part-time*	186 (22.0)	6 (2.4)	82 (25.6)	98 (36.0)
*Full-time*	317 (37.4)	75 (29.4)	106 (33.1)	136 (50.0)
Living situation, *n* (%)				
*Alone*	144 (17.0)	30 (12.2)	45 (14.1)	68 (25.0)
*Supported (parent(s))*	364 (43.0)	27 (10.6)	250 (78.1)	87 (32.0)
*Individual (partner, family)*	339 (40.0)	197 (77.3)	25 (7.8)	117 (43.0)
DUP (in weeks)^a^				
*M (SD)*	29.66 (60.58)	32.01 (64.43)	32.66 (57.22)	23.36 (60.24)
*Median (IQR)*	6 (6–20)	6 (6–18.8)	9 (9–29)	4 (4–12)
Diagnosis, *n* (%)^b^				
*Schizophrenia*	408 (48.3)	108 (42.35)	179 (55.94)	121 (44.49)
*Schizoaffective*	46 (5.4)	11 (4.31)	17 (5.31)	18 (6.62)
*Depressive*	129 (15.3)	50 (19.61)	34 (10.63)	45 (16.54)
*Manic, bipolar*	132 (15.6)	48 (18.82)	46 (14.38)	38 (13.97)
*Delusional disorder*	43 (5.1)	11 (4.31)	18 (14.38)	14 (5.15)
*NOS, other, and uncertain* ^c^	87 (10.3)	27 (10.59)	26 (8.13)	34 (12.50)
Affective psychosis, *n* (%)	261 (30.81)	98 (38.43)	80 (25.00)	83 (30.51)
Country, *n* (%)				
*United Kingdom*	180 (21.25)	63 (24.71)	70 (21.88)	47 (17.28)
*Holland*	167 (19.72)	38 (14.90)	66 (20.62)	63 (23.16)
*Spain*	130 (15.35)	31 (12.16)	50 (15.63)	49 (18.01)
*France*	45 (5.31)	19 (7.45)	12 (3.75)	14 (5.15)
*Italy*	149 (18.59)	26 (10.20)	53 (16.56)	70 (25.73)
*Brazil*	176 (20.79)	78 (30.59)	69 (21.56)	29 (10.66)

a We excluded *n* = 82 (9.7%) participants from the between-class comparisons on time to treatment, thereof *n* = 61 due to missing values and *n* = 21 for outlier status (values above 3 SDs for DUP); *n* = 13 (5.1%) in class 1, *n* = 27 (8.4%) in class 2, and *n* = 41 (15.4%) in class 3;

b missing diagnosis: *n* = 2 (0.2%);

c NOS = not otherwise specified psychosis; this category includes uncertain diagnosis and cases where the algorithm could not derive diagnosis.

#### Class 1: Older, often unemployed, with ethnic minority group status, often living with their partner or family

Patients in class 1 were on average 34.73 years old (SD = 11.17), with slightly more women (60.4%). A high proportion in this class was from ethnic minority groups (63.9%). One-third (35.6%) had no school-leaving qualification or elementary education, and 12.9% held a university degree. They were often unemployed (68.2%) and lived predominantly with their partner or other family members besides parents (77.3%).

#### Class 2: Younger men, often unemployed, with more cannabis use, living with their parents

This was the largest class with comparably younger age (*M* = 26.36 years, SD = 8.32), predominantly male (97.8%) patients, and a higher proportion of cannabis use (37.8%). Almost half (41.2%) were currently unemployed. Patients had predominantly secondary education (85.3%) and lived with their parent(s) (78.1%).

#### Class 3: White, rather employed, and with a higher educational level

Patients in class 3 were more often female (64%), and predominantly White (93.4%), with the lowest proportion (8.1%) of cannabis use. Patients were more educated, with 64.4% having completed secondary education and 35.3% with a university degree. They were mostly in part-time employment/students (36.0%) or full-time employment (50.0%).

In Cox proportional hazard regression analysis, class 2 showed a longer time to treatment compared with class 3 (HR = 1.27; 95% CI: 1.07–1.51, *p* = .007). No significant differences in DUP were found between class 1 and class 2 (HR = 0.93, 95% CI 0.91–1.28, *p* = .405). There was a modest difference between class 1 and 3 (HR = 1.18, 95% CI 0.98–1.42, *p* = .073). The respective Kaplan–Meier plot is shown in Figure S7 in the Supplementary Material. Proportional hazard assumption, inspected via Schoenfeld residuals, was given *χ*^2^(2, *N* = 765) = 4.90, *p* = .086, indicating that the hazard ratio is constant over time.

## Discussion

### Main findings

The current study identified three classes of configurations of social and substance use-related risks in two large cohorts of patients with psychotic disorders, in line with our first aim. In both samples, class 2, characterized by younger age, male sex, high proportions of cannabis use, and supported living situation, had longer DUP compared with class 3, characterized by White ethnicity, higher levels of educational attainment, and employment. Still, there were substantial differences between the samples: the patients in class 2 in GROUP were more often unemployed and had lower levels of educational attainment than those in EU-GEI. In GROUP, the patients in class 3 were predominantly living alone, which was less frequent in that class in EU-GEI. Moreover, the third identified class was unique in each sample. In the GROUP sample with enduring psychosis, another class emerged with the shortest DUP, characterized by female sex, predominantly White ethnicity and no cannabis use, although higher unemployment. By contrast, the third class in the EU-GEI FEP sample was characterized by ethnic minority group status, current unemployment, and living with a partner or family, with a nonsignificant trend toward longer DUP, compared to class 3. Overall, this aligns with our hypothesis that subpopulations that index clustering for several risk factors for social and substance use-related risks can experience longer DUP.

### Risk configurations for longer DUP

Risk factors that have so far been independently associated with longer DUP contributed to the most unfavorable configuration: the population of younger, male, unemployed patients in supported living who use cannabis showed prolonged DUP. Hence, this subpopulation shares most risk factors previously associated with longer DUP, such as unemployment (Limbu et al., 2025; Morgan et al., 2006; Qiu et al., 2019; Reichert & Jacobs, 2018), lower SES (Fordham et al., 2023; Peralta et al., 2005), and male sex (Apeldoorn et al., 2014; Skrobinska et al., 2024). Overall, this aligns with prior work conducted in a cohort of patients with psychosis from the United States, where the more disadvantaged classes in terms of social position and first-contact experienced a longer DUP (van der Ven et al., 2022). However, direct comparisons are challenging because they investigated time to first contact and time to specialized care separately, and the United States has a vastly different health care system. Furthermore, the class with predominantly White individuals in the study in the United States mainly diverged from the other identified classes and experienced the shortest time from onset to first contact (van der Ven et al., 2022). This echoes our results, as White ethnicity was most prevalent in class 3 in both samples and class 1 in GROUP, showing shorter DUP.

Our results underline the importance of interpreting social and substance use-related risks within their broader context. Prolonged DUP was only observed in the presence of specific configurations of multiple risk factors (class 2), whereas other configurations were not associated with longer DUP. For example, class 1 in GROUP – with the shortest DUP – was characterized by female sex, White ethnicity, and the absence of cannabis use, but showed relatively high rates of unemployment. This specific configuration may create intersections that buffer the negative effect of unemployment through other financial and social resources, such as higher levels of social contacts (Reininghaus et al., 2008). Contrary to previous results suggesting that living with family members is associated with shorter DUP compared to living alone (Compton et al., 2011), in our study, living with parents was part of risk configurations (class 2), whereas the class with shorter DUP (class 3) lived alone more frequently. Similarly, the association of recent cannabis use and DUP differed in accordance with risk configurations: while cannabis use was associated with prolonged DUP in class 2, and the absence of cannabis use was linked to shorter DUP in class 1 in GROUP, class 3 in GROUP still exhibited higher levels of cannabis use and a shorter DUP, potentially buffered by higher SES. In addition, the association between attained education and DUP remains equivocal. Although prior work in the Brazilian EU-GEI subsample (Shuhama et al., 2021) and other studies (Skrobinska et al., 2024; Souaiby et al., 2019; Takizawa et al., 2021) reported an association between lower levels of educational attainment and longer DUP, prior work in GROUP did not (Apeldoorn et al., 2014). Notably, lower educational attainment in our study was most prevalent in classes with younger age, who may not yet have completed college.

Prolonged DUP has been associated with higher symptom severity and a lower chance of remission in psychotic disorders (Howes et al., 2021). Thus, the question remains whether risk configurations of male sex, unemployment, cannabis use, and supported living may not only predict longer DUP, but may also directly index risk for poorer overall prognosis. Identified classes with cumulated adverse social and behavioral factors may cause treatment delays and simultaneously prevent a more favorable course and long-term outcome in psychosis. For example, lower levels of educational attainment and substance use have been previously reported to be associated with nonadherence and treatment dropout (Leclerc, Noto, Bressan, & Brietzke, 2015), and cannabis use with relapse (Hasan et al., 2020). Therefore, treatment outcomes might be shaped by the same upstream social and substance use-related factors that impact DUP, creating a dual pathway that increases health disparities. More generally, the complex interplay of how adverse social experiences may co-occur, impact, and intersect with each other over time to impact DUP remains to be established. Ignoring or neglecting the effects of social determinants on DUP and the interaction with treatment outcomes might inadvertently augment social and ethnic inequalities in health.

There is an indication that a difference in median DUP of 4–5 weeks (between class 2 and class 3) might be clinically relevant: It has been previously reported that patients with a DUP of 4 weeks compared to 1 week may have up to 20% more severe symptoms (Howes et al., 2021). Thus, subpopulations might need alternative, more targeted pathways to, and lower thresholds for accessing care, as shorter DUP accelerates functional recovery and seems critical for maximizing the effects of early intervention (Hazan et al., 2025). Some populations, for example, ethnic minority groups, may be less likely to receive early intervention for psychosis (Schlief et al., 2023), and the coordinated specialized care actually received may be less effective, as has been shown for patients with lower SES (Bennett & Rosenheck, 2021). Early intervention for psychotic disorders may be improved if delivered to these populations more effectively. Addressing the underlying social and behavioral factors might not only improve treatment delays, but also illness trajectories down the line. Future studies should integrate a broader range of social determinants and exposures, for example, childhood adversity (Kirkbride et al., 2024), and associated neighborhood characteristics (Oluwoye et al., 2024), to evaluate if any marginalized groups with at-risk configurations exist and identify potential targets for intervention. Programs that aim to reduce DUP should evaluate differential effects, which were previously highlighted as a research gap (Murden, Allan, Hodgekins, & Oduola, 2024), to move toward more equitable interventions in public mental health (Reininghaus et al., 2024).

### Strengths and limitations

This study investigated two large samples with patients from different countries and sites, and applied a consistent methodological approach to enhance the robustness of findings.

The current study has several limitations. The cohorts differ in recruitment periods and encompass geographic heterogeneity, spanning multiple countries with diverse healthcare systems and sociocultural contexts, potentially introducing unmeasured (context-specific) confounders that are difficult to disentangle. EU-GEI included patients with a first episode of both affective and nonaffective psychosis, whereas GROUP comprised a naturalistic cohort of patients with enduring, nonaffective psychosis. To partially address this, we excluded outliers in GROUP with long illness durations.

Further, the sociodemographic factors were assessed at the time of study participation in GROUP, and some are modifiable and can change over time. For example, individuals with psychosis tend to lose their jobs at the beginning of the disorder (Rinaldi et al., 2010). In EU-GEI, the variables were assessed for the time of onset of psychotic symptoms. This reduces possible interferences and strengthens the results.

Next, the representation of each ethnic minority group did not allow for the performance of disaggregated analysis as suggested and done by other studies (Schoer, Huang, & Anderson, 2019; van der Ven et al., 2022). Further research is needed with participants from diverse societies and populations.

Moreover, DUP is susceptible to recall bias, especially for patients with a long DUP and/or a longer duration of illness, as the disorder might affect long-term recall. On top of that, DUP might be less accurate in GROUP, as the onset of the disorder was, on average, a longer time ago. To reduce measurement errors, the projects implemented structured assessments and training (see Supplementary Material 1).

Last, the DUP contains many ties, that is, multiple events (treatment start) at the same time point, especially in short survival times. This could affect the parameter estimates in the survival analysis because the partial likelihood for tied events cannot be directly calculated but is corrected for, which might underestimate the resulting hazard ratio. We used Efron’s approximation method, which is considered the best procedure for handling ties (Scheike & Sun, 2007).

## Conclusion

The current study revealed subpopulations of patients with psychotic disorders that index configurations of social and substance use-related risks, indicating risk for prolonged DUP. Identifying marginalized subpopulations that experience longer DUP might shed light on blind spots in access to mental health care. Future studies should identify differential key aspects in help-seeking and access to care that contribute to delayed treatment initiation in FEP for subpopulations, refining targeted early intervention programs for DUP while addressing health inequalities.

## Supporting information

10.1017/S0033291726103791.sm001 Edelhoff et al. supplementary material Edelhoff et al. supplementary material

## Supplementary material

The supplementary material for this article can be found at http://doi.org/10.1017/S0033291726103791.
