# Supplementary material for: Clusters of social and substance use-related risks are associated with the duration of untreated psychosis
Source: Psychol Med. 2026 May 5;56:e126. doi: 10.1017/S0033291726103791 (PMC13161825; doi:10.1017/S0033291726103791)
Supplement: Edelhoff et al. supplementary material [file S0033291726103791sup001.docx]

# Supplements

Supplement 1. Description of interview training and DUP assessment.

Figure S1. Missing values in the total sample in GROUP (n = 1119).

Figure S2. Missing data matrix for GROUP.

Figure S3. Missing values in the total sample in EU-GEI (n = 1130).

Figure S4. Missing data matrix for EU-GEI.

Table S1. 2-class solution in GROUP.

Table S2. 2-class solution in EU-GEI.

Figure S5. Kaplan-Meier plot for DUP antipsychotic treatment in GROUP (n = 676).

Figure S6. Kaplan-Meier plot for DUP contact to mental health services in GROUP (n = 671).

Figure S7. Kaplan-Meier plot for DUP antipsychotic treatment in EU-GEI (n = 765).

**Supplement 1. Description of interview training and DUP assessment.**

The interviewers in GROUP and EU-GEI were psychologists, psychiatrists, nurses, research assistants, and PhD students. They completed a structured three-day on site interview training and received written procedure manuals and video instructions. New interviewers entering the project were trained in didactic sessions, observation, and supervised practice. Moreover, all interviewers completed ongoing quarterly reliability training. Diagnostic consensus was reached with independent psychiatrists.

Three sites in GROUP operationalized the start of psychotic symptoms according to the criteria of the Comprehensive Assessment of Symptoms and History (CASH) (Andreasen, Flaum, & Arndt, 1992) and one site used the Schedules of Clinical Assessment of Neuropsychiatry (SCAN 2.1) (Wing, Cooper, & Sartorius, 1974). The Life Chart Schedule (Sartorius, Gulbinat, Harrison, Laska, & Siegel, 1996) was used to collect information from patients, parent(s) and clinical records. If treatment started before the onset of psychosis, i.e., in the prodromal phase, the DUP was set to 0. GROUP assessed only the month/year of onset and treatment, therefore, patients with a DUP with up to 4 weeks were recorded with 0 months. The reliability was fair to excellent for ratings of duration of experience with an interclass correlation coefficient (ICC) between 0.53 to 0.99 (Korver et al., 2012).

EU-GEI operationalized the start of the psychotic symptoms as hallucinations, delusions, or thought disorder rated as moderate-severe (4) or higher on the Positive and Negative Syndrome Scale (PANSS)(Kay, Fiszbein, & Opler, 1987). Case notes from the patient files were used where available to confirm the dates. The ratings of the 105 core researchers for the positive symptoms scale showed substantial interrater reliability with κ = 0.79 (Gayer-Anderson et al., 2020).

References:

Andreasen, N. C., Flaum, M., & Arndt, S. (1992). The Comprehensive Assessment of Symptoms and History (CASH). An instrument for assessing diagnosis and psychopathology. *Arch Gen Psychiatry, 49*(8), 615-623. doi:10.1001/archpsyc.1992.01820080023004

Gayer-Anderson, C., Jongsma, H. E., Di Forti, M., Quattrone, D., Velthorst, E., de Haan, L., . . . Morgan, C. (2020). The EUropean Network of National Schizophrenia Networks Studying Gene-Environment Interactions (EU-GEI): Incidence and First-Episode Case-Control Programme. *Soc Psychiatry Psychiatr Epidemiol, 55*(5), 645-657. doi:10.1007/s00127-020-01831-x

Kay, S. R., Fiszbein, A., & Opler, L. A. (1987). The positive and negative syndrome scale (PANSS) for schizophrenia. *Schizophr Bull, 13*(2), 261-276. doi:10.1093/schbul/13.2.261

Korver, N., Quee, P. J., Boos, H. B., Simons, C. J., de Haan, L., & investigators, G. (2012). Genetic Risk and Outcome of Psychosis (GROUP), a multi-site longitudinal cohort study focused on gene-environment interaction: objectives, sample characteristics, recruitment and assessment methods. *Int J Methods Psychiatr Res, 21*(3), 205-221. doi:10.1002/mpr.1352

Sartorius, N., Gulbinat, W., Harrison, G., Laska, E., & Siegel, C. (1996). Long-term follow-up of schizophrenia in 16 countries. A description of the International Study of Schizophrenia conducted by the World Health Organization. *Soc Psychiatry Psychiatr Epidemiol, 31*(5), 249-258. doi:10.1007/BF00787917

Wing, J. K., Cooper, J. E., & Sartorius, N. (1974). *Measurement and classification of psychiatric symptoms; An instruction manual for the PSE and Catego program*. Oxford, England: Cambridge U Press.

## Figure S1. Missing values in the total sample in GROUP (n = 1119).


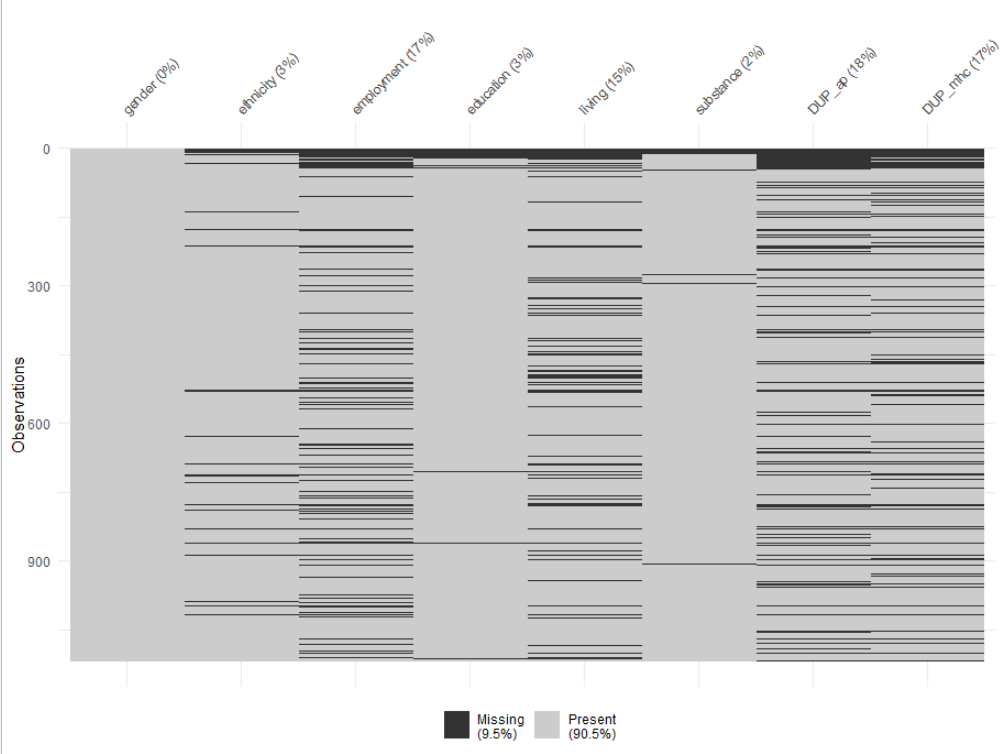


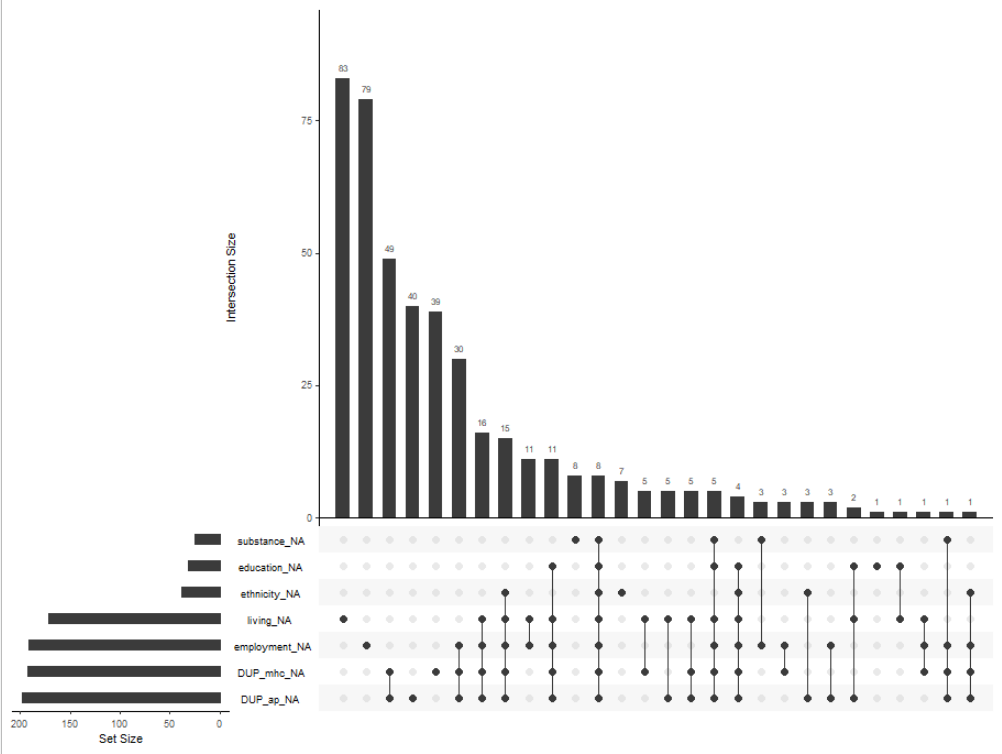


## Figure S2. Missing data matrix for GROUP.


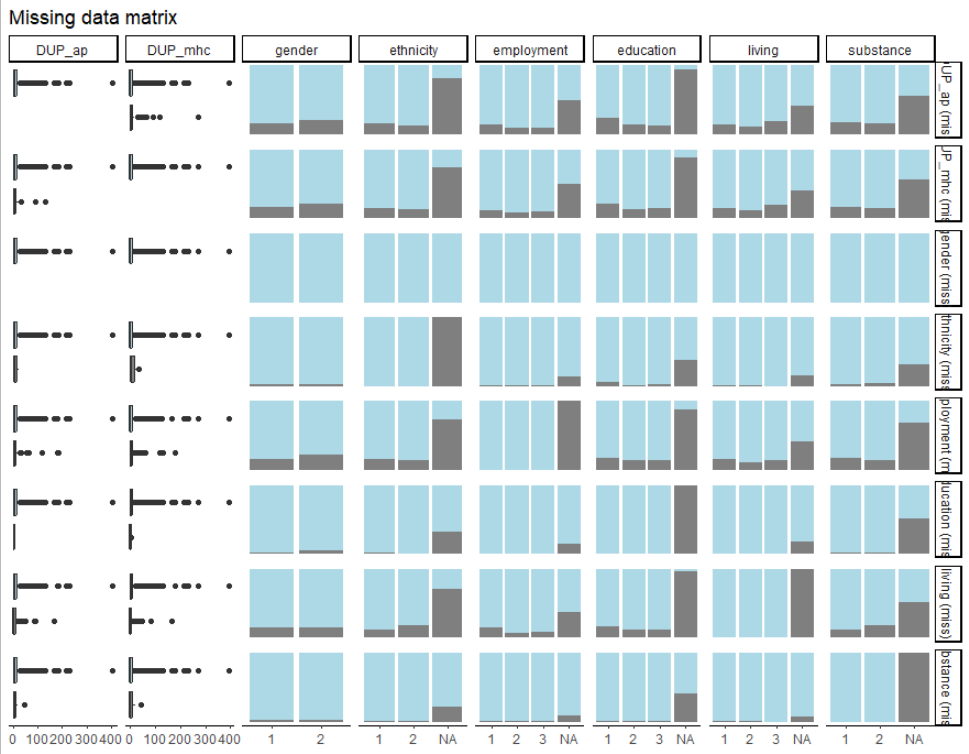


## Figure S3. Missing values in the total sample in EU-GEI (n = 1130).


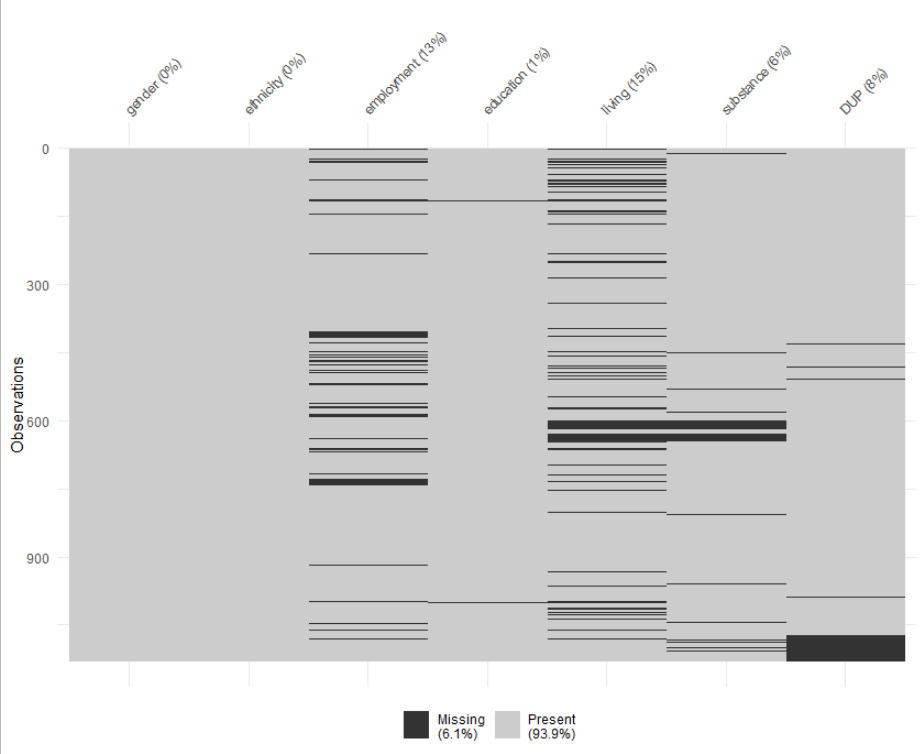


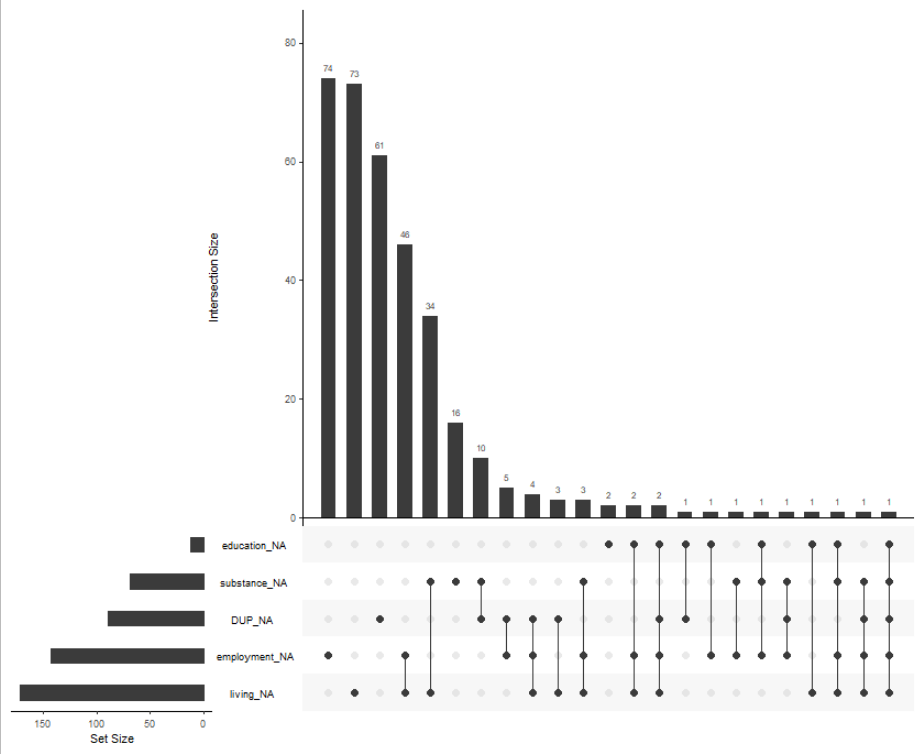


## Figure S4. Missing data matrix for EU-GEI.


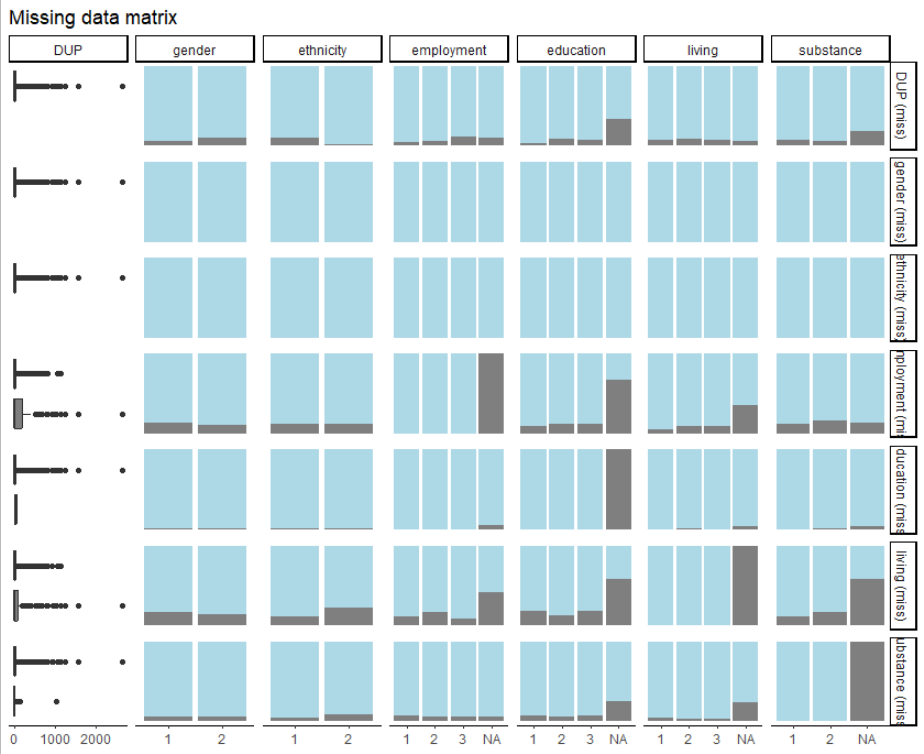


## Table S1. 2-class solution in GROUP

| **Variable** | **Total sample**  (n = 780) | **Class 1**  (n = 196, 25.13%) | **Class 2**  (n = 584, 74.87%) |
| --- | --- | --- | --- |
| Age (in years), M (SD) | 26.84 (7.18) | 30.53 (8.27) | 25.60 (6.32) |
| Sex, male, n (%) | 606 (77.7) | 63 (32.14) | 543 (92.98) |
| Ethnicity, White, n (%) | 623 (79.9) | 185 (94.39) | 438 (75.00) |
| Cannabis use, yes, n (%) | 291 (37.3) | 2 (1.02) | 289 (49.49) |
| Highest education, n (%)  *No education/elementary*  *Secondary*  *University* | 104 (13.3)  577 (74.0)  99 (12.7) | 4 (2.04)  113 (57.65)  79 (40.31) | 100 (17.12)  464 (79.45)  20 (3.42) |
| Employment status, n (%)  *Unemployed*  *Part-time*  *Full-time* | 338 (43.3)  230 (29.5)  212 (27.2) | 73 (37.24)  64 (32.65)  59 (30.10) | 265 (45.38)  166 (28.42)  153 (26.20) |
| Living situation, n (%)  *Alone*  *Supported (parent(s), sheltered)*  *Individual (partner, family)* | 265 (34.0)  433 (55.5)  82 (10.5) | 57 (29.08)  73 (37.24)  66 (33.67) | 208 (35.62)  360 (61.64)  16 (2.74) |
| Class (in the 3-class solution)  Class 1  Class 2  Class 3 |  | 129  4  63 | 4  470  110 |

## Table S2. 2-class solution in EU-GEI

| **Variable** | **Total sample**  (n = 847) | **Class 1**  (n = 502, 59.27%) | **Class 2**  (n = 345, 40.73 %) |
| --- | --- | --- | --- |
| Age (in years), M (SD) | 31.21 (10.74) | 34.92 (10.97) | 25.83 (7.71) |
| Sex, male, n (%) | 512 (60.5) | 233 (46.4) | 279 (80.9) |
| Ethnicity, White, n (%) | 550 (64.9) | 304 (60.6) | 246 (71.3) |
| Cannabis use, yes, n (%) | 176 (20.8) | 60 (12.0) | 116 (33.6) |
| Highest education, n (%)  *No education/elementary*  *Secondary*  *University* | 139 (16.4)  578 (68.2)  130 (15.4) | 96 (19.1)  283 (56.4)  123 (34.5) | 43 (12.5)  295 (85.5)  7 (2.0) |
| Employment status, n (%)  *Unemployed*  *Part-time*  *Full-time* | 344 (40.6)  186 (22.0)  317 (37.4) | 213 (42.4)  78 (15.5)  211 (42.0) | 131 (38.0)  108 (31.3)  106 (30.7) |
| Living situation, n (%)  *Alone*  *Supported (parent(s))*  *Individual (partner, family)* | 144 (17.0)  364 (43.0)  339 (40.0) | 120 (23.9)  49 (9.8)  333 (66.3) | 24 (7.0)  315 (91.3)  6 (1.7) |
| Class (in the 3-class solution)  Class 1  Class 2  Class 3 |  | 255  43  204 | 0  277  68 |

## Figure S5. Kaplan-Meier plot for DUP antipsychotic treatment in GROUP (n = 676).


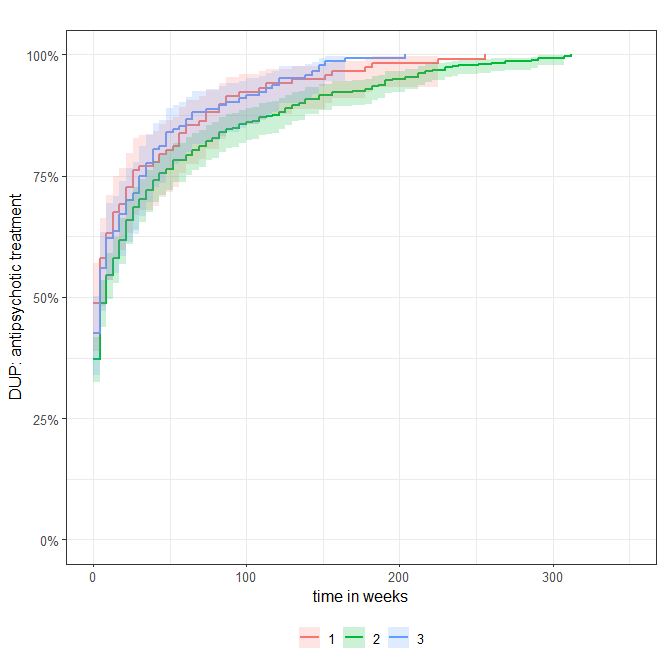


*Notes*. Class 1 = White women without cannabis use and higher unemployment; class 2 = Younger men, with lower educational attainment, higher unemployment, cannabis use and supported living situation; class 3 = White men, employed, with higher educational attainment, often living alone.

## Figure S6. Kaplan-Meier plot for DUP contact to mental health services in GROUP (n = 671).


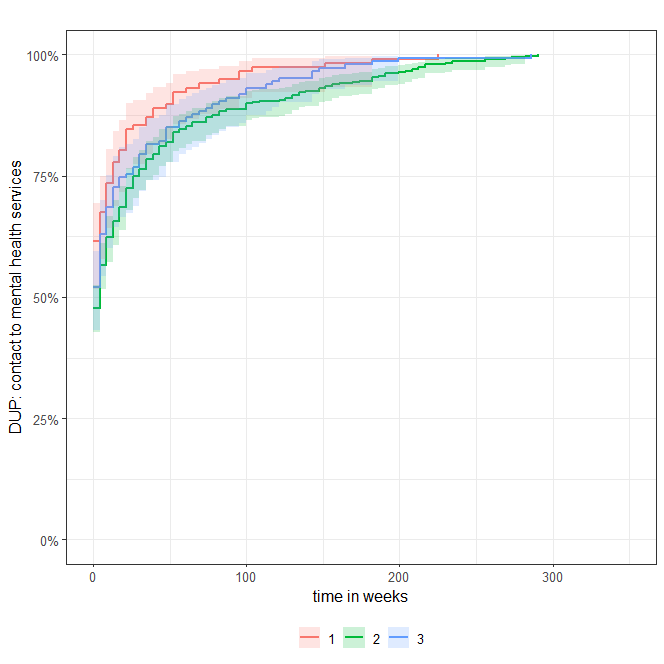


*Notes*. Class 1 = White women without cannabis use and higher unemployment; class 2 = Younger men, with lower education, higher unemployment, cannabis use and supported living situation; class 3 = White men, employed, with higher educational level, often living alone.

## Figure S7. Kaplan-Meier plot for DUP antipsychotic treatment in EU-GEI (n = 765).


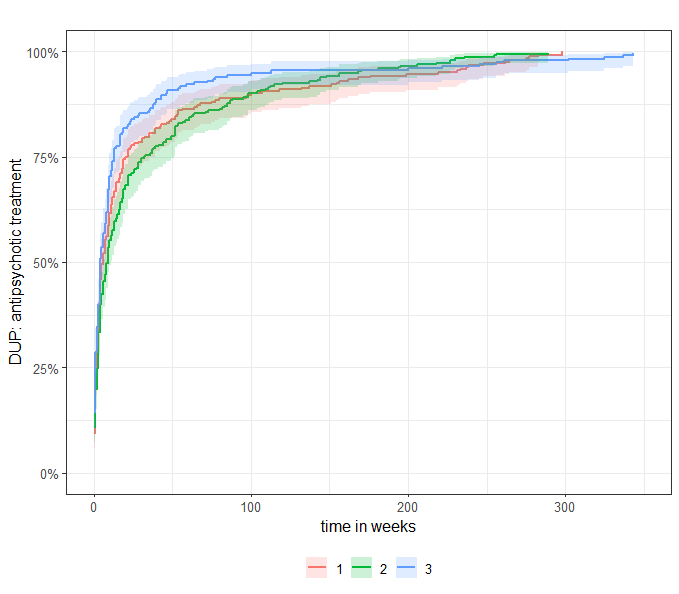


*Notes*. Class 1 = Older, often unemployed, with ethnic minority group status, often living with their partner or family; class 2 = Younger men, often unemployed, with more cannabis use, living with their parents; class 3 = White, rather employed and with higher educational level.
